# Maternal diabetes independent of BMI is associated with altered accretion of adipose tissue in large for gestational age fetuses

**DOI:** 10.1371/journal.pone.0268972

**Published:** 2022-05-31

**Authors:** Penny Lam, Brendan J. Mein, Ronald J. Benzie, John T. Ormerod, Kristy P. Robledo, Emily J. Hibbert, Ralph K. Nanan

**Affiliations:** 1 Christopher Kohlenberg Department of Perinatal Ultrasound, Nepean Hospital, Penrith, New South Wales, Australia; 2 School of Mathematics and Statistics, University of Sydney, Sydney, New South Wales, Australia; 3 ARC Centre of Excellence for Mathematical and Statistical Frontiers, The University of Melbourne, Parkville, Victoria, Australia; 4 NHMRC Clinical Trials Centre, University of Sydney, Sydney, New South Wales, Australia; 5 Department of Endocrinology and Diabetes, Division of Medicine, The University of Sydney Nepean Clinical School, University of Sydney, Penrith, New South Wales, Australia; 6 Discipline of Paediatrics and Child Health, University of Sydney, Sydney, New South Wales, Australia; 7 Charles Perkins Centre–Nepean Clinical School, University of Sydney, Penrith, New South Wales, Australia; University of Guelph Ontario Agricultural College, CANADA

## Abstract

**Aim:**

To analyse the effects of maternal diabetes mellitus (DM) and body mass Index (BMI) on central and peripheral fat accretion of large for gestational age (LGA) offspring.

**Methods:**

This retrospective study included LGA fetuses (n = 595) with ultrasound scans at early (19.23 ± 0.68 weeks), mid (28.98 ± 1.62 weeks) and late (36.20 ± 1.59 weeks) stages of adipogenesis and measured abdominal (AFT) and mid-thigh (TFT) fat as surrogates for central and peripheral adiposity. Women were categorised according to BMI and DM status [pre-gestational (P-DM; n = 59), insulin managed (I-GDM; n = 132) and diet managed gestational diabetes (D-GDM; n = 29)]. Analysis of variance and linear regressions were applied.

**Results:**

AFT and TFT did not differ significantly between BMI categories (normal, overweight and obese). In contrast, AFT was significantly higher in pregnancies affected by D-GDM compared to non-DM pregnancies from mid stage (0.44 mm difference, p = 0.002) and for all DM categories in late stage of adipogenesis (≥ 0.49 mm difference, p < 0.008). Late stage TFT accretion was higher than controls for P-DM and I-GDM but not for D-GDM (0.67 mm difference, p < 0.001; 0.49 mm difference, p = 0.001, 0.56 mm difference, p = 0.22 respectively). In comparison to the early non-DM group with an AFT to TFT ratio of 1.07, the I-GDM group ratio was 1.25 (p < 0.001), which normalised by 28 weeks becoming similar to control ratios.

**Conclusions:**

DM, independent of BMI, was associated with higher abdominal and mid-thigh fat accretion in fetuses. Use of insulin improved central to peripheral fat ratios in fetuses of GDM mothers.

## Introduction

The worldwide increase in obesity and diabetes mellitus (DM) continues to place immense strain on health systems [1, 2]. The development of obesity and DM has been linked to early exposures during fetal life [3, 4]. Several epidemiological studies have identified long-term metabolic consequences in offspring of mothers with obesity and DM [5, 6]. These metabolic consequences include an increased risk for metabolic syndrome, type 2 DM (T2DM), obesity and cardiovascular disease (CVD) [7]. This transgenerational sequence of metabolic disorders might explain the global increase in obesity and DM.

Closer investigation of maternal DM and obesity shows a strong link to fetal overgrowth resulting in large for gestational age (LGA) babies [8, 9]. Interestingly, LGA babies are more prone to metabolic disorders in childhood leading to an increased risk of DM and obesity later in life [10]. The metabolic differences in mothers with DM or obesity may alter the intrauterine environment resulting in fetal overgrowth [11].

Disproportionate overgrowth of the abdomen is commonly found in fetuses of DM mothers [12, 13]. This is important as abdominal subcutaneous and visceral fat in children are positively associated with cardiometabolic risk factors [14, 15]. Abdominal fat also appears to be the tissue implicated in metabolic health and in the development of CVD and T2DM [16], whereas peripherally distributed (lower body) fat may be protective of metabolic disease [16, 17].

An ultrasound method to assess fetal subcutaneous fat, in a population of average for gestational age neonates, has already been establish. Several studies have demonstrated a strong association between subcutaneous fat and birthweight with increased fetal fat accretion found in mothers with GDM [18–20]. O’Connor et al. states that subcutaneous fat in the abdomen and thigh improves the predictive power for weight estimation and provided a reference chart of thigh fat measurements between 28–37 weeks of gestation. Larciprete et al. and Aksoy et al., in a similar cohort of normal BW neonates, found GDM mothers increased subcutaneous fat in offspring [19, 20]. Normal references ranges were documented for healthy pregnancies from 20–40 weeks of gestation by Larciprete et al. [19].

Since GDM in a normal birthweight population has already demonstrated an increase in fetal subcutaneous fat [18–20], we have specifically evaluated LGA fetuses, which poses a risk for metabolic syndrome and examined the effect different types of DM can have on the fetus. As stated previously, LGA fetuses are commonly found in obese or DM mothers so we firstly explored the differences between these groups before observing differences in DM subtypes. Cioffi et al. demonstrated that the distribution of fat changes in adolescents [21], therefore we will further assess the patterns of fat distribution in fetuses effected by maternal DM.

The gestational age groups chosen for the study were based on the time of adipose tissue formation. Given that adipose tissue begins to form during the 2^nd^ trimester, with 90% of fat accumulating in the last 10 weeks of the 3^rd^ trimester [22], we examined fetal subcutaneous fat during the 2^nd^ and 3^rd^ trimesters of pregnancy. Ultrasound scans between gestational weeks of 17–22 weeks, 25–32 weeks and 33–40 weeks represented early, middle, and late stages of fetal fat development, respectively. Subcutaneous tissue of the abdomen and thigh, representing central and peripheral fat was measured on an existing dataset of routinely taken ultrasound images for LGA fetuses.

The aim of this study was to compare subcutaneous fat accretion patterns in LGA fetuses between pregnancies affected by maternal obesity and maternal DM. Here we hypothesised that fetuses affected by maternal DM are likely to have increased subcutaneous fat accretion with higher AFT to TFT ratios.

## Material and methods

This was a retrospective study in which ultrasound scans of 595 women with LGA babies from January 2006 to December 2017 were assessed. LGA was defined by neonatal birth weight greater than the 90^th^ centile of the expected weight at a given gestational age [23, 24]. Before commencing the study, approval was granted by the APOLLO Ethics Review Panel from the Nepean Blue Mountains Area Health District Human Research Ethics Committee (Study 16/95). The study was exempted from full ethical review and informed consent from the participants was not required. The data was also de-identified at the time of analysis.

### Study population

Data on 47134 participants over 11 years was retrieved from the Nepean Hospital Obstetrix database. LGA deliveries from 34 weeks gestation or greater were selected. Participants were women with singleton pregnancies with no disorders affecting fetal growth, other than DM or obesity, and had three consecutive scans conducted in the Department of Perinatal Ultrasound, Nepean Hospital. Twins and fetuses with structural or chromosomal abnormalities were excluded from the study. The scans were performed by trained professionals in fetal ultrasonography using GE Voluson ultrasound machines (General Electric Healthcare). The gestational age was calculated according to the patient’s last menstrual period (LMP) or first trimester dating ultrasound. The estimated due date calculated from the dating scan was preferred if the LMP dates differed by more than 4 days.

Participants were categorised according to BMI and DM status. Self-reported pre-pregnancy BMI data was collected in the first trimester and validated at the first antenatal clinic appointment. The BMI groups were divided into three categories, normal weight (18.5 to < 25 kg/m^2^), overweight (25 to < 30 kg/m^2^), obese (≥ 30.0 kg/m^2^). As standard practice, all participants were tested for gestational diabetes mellitus (GDM) between 24–28 weeks gestation in accordance with the Australasian Diabetes in Pregnancy Society (ADIPS) guideline for diagnosis of GDM and DM in pregnancy [25]. The pregnancy Oral Glucose Tolerance Test (OGTT) involved a fasting plasma glucose test followed by sampling of plasma glucose 1 hour and 2 hours after a 75g oral glucose load. Participants prior to 2017 were classified as having GDM if readings were at or above the cut-off values as indicated in the 1998 ADIPS consensus guideline [25]; ADIPS guidelines published in 2014 were implemented thereafter [26]. Testing was performed at an earlier stage of the pregnancy for participants at higher risk for GDM. Specific treatments were given to participants according to their glucose levels on the OGTT and their ability to regulate blood glucose levels, as per usual practice. All participants with DM were referred for a group education session with a diabetes educator and dietitian for blood glucose monitoring and diet and activity plan modifications. Participants with lower glucose levels on their OGTT were managed with diet and activity alone, unless self-reported blood glucose monitoring showed levels outside the target range, in which case they were started on insulin. Participants with higher range glucose results were treated with insulin if glucose levels remained high after dietary and activity modification. Diabetes study groups were arranged according to the type of diabetes: pre-gestational DM (P-DM), GDM insulin managed at any point during the pregnancy (I-GDM) and GDM diet managed (D-GDM). Participants who had normal OGTT readings were the control group.

### Ultrasound measurements

Fetal fat was assessed from standard growth images of the abdominal circumference (AC) and femur length (FL), which were previously obtained and stored on a dedicated obstetric ultrasound reporting system (ViewPoint software V5). Ultrasound images were captured using General Electric Voluson E8 and E10 ultrasound machines with 4–8 MHz wideband curvilinear abdominal transducers (RAB6-RS; General Electric Medical Systems Austria). All studied images were reviewed by a single expert sonographer to ensure images met the criteria for analysis. The abdominal fat thickness (AFT), which included skin and subcutaneous tissue, was obtained from the AC image by measuring the clearest, not compressed portion of the wall (adapted from Higgins et al. [27]). The mid-thigh thickness (TFT) was taken at the middle point of the thigh (± 2 cm) from the FL image and included skin and subcutaneous tissue [18]. The measured area was also not compressed by structures such as the uterine wall or other fetal body parts. Measurements of the abdomen and thigh fat were performed twice, and a mean was recorded. Sonographer, PL, reviewed fetal fat at 3 time points ranging between 17–22 weeks, 25–32 weeks and 33–40 weeks of gestation with a minimum of 2 weeks difference between each time point; the time points represented early, middle, and late stages of fetal fat development, respectively.

Comparisons of fetal fat measurements were made between BMI and DM groups. An abdomen to mid-thigh fat ratio was also calculated to assess the distribution of central versus peripheral adipose tissue in the body. To assess for reproducibility, fetal subcutaneous fat from a random selection of 30 participants was measured by an expert sonographer (BM), who was blinded to the previous measurements. The same images were measured by PL to assess for intra-observer variability. Perinatal outcomes of gestational age at delivery, birth weight, gender, smoking status, and parity were also reviewed.

### Statistical analysis

Baseline characteristics were compared using a chi-squared test for categorical data, and either a 2-sample t-test or a Mann-Whitney U test for normal or non-normal continuous data, respectively.

For AFT and TFT over time, a linear model was fit. A time effect, BMI group effect, diabetic status (control versus diabetes) and an interaction term between (A) DM status and time and (B) BMI group and time were fit in this model. As the interaction of BMI and time was not significant, this was removed from the final model; the main effect of BMI and time alone remained in the model. The interaction effect with DM status and time was of main interest. Similar models were fit with (A), replaced with DM subtypes and where the ratio AFT divided by TFT was used as the response. ANOVA was used to determine the effect of each categorical variable. Normal weight was set as the baseline for the BMI category and participants without DM (control) for the DM category and DM subtypes. Data was expressed as means (95% CI).

The rate of abdominal fat change and the rate of thigh fat change were derived via linear regression with different combinations of the 3 time points, early (E), mid (M) and late (L). This results in 3 different approaches to estimating these rates (E-M), (M-L) and (E-M-L). Similar linear models to those described above were fit with rate of abdominal fat change and the rate of thigh fat change used as the response. Data was expressed as mean / week (95% CI).

Intraclass correlation was used to determine the degree of inter-operator and intra-operator agreement.

## Results

**Fig 1:**
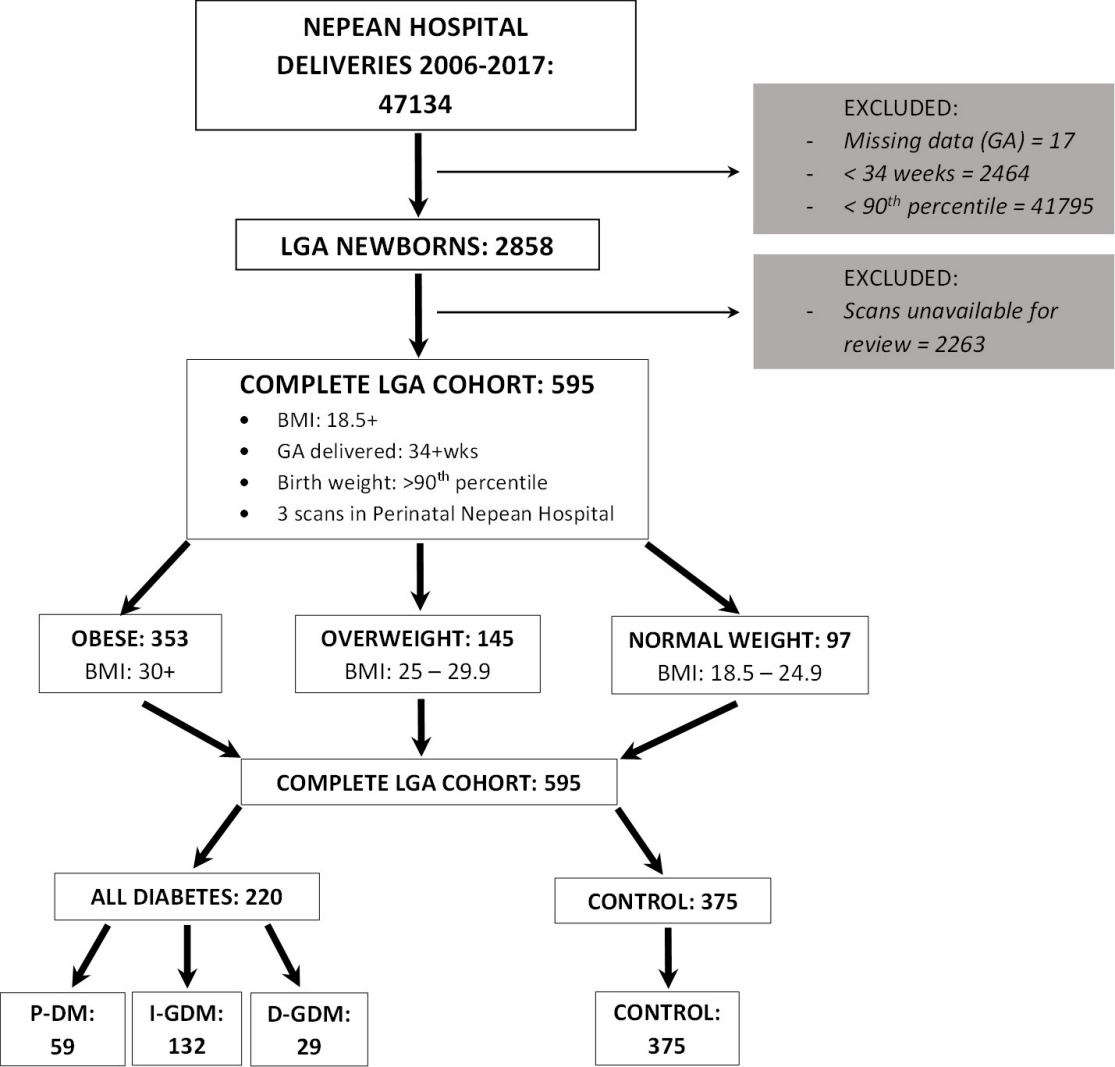
Study flowchart. Selection of participants based on prescribed criteria. LGA, large for gestational age; GA, gestational age; DM, diabetes mellitus; P-DM, pre-gestational DM; I-GDM, GDM insulin managed and D-GDM, GDM diet managed.

Data on 47134 deliveries was retrieved from the Obstetrix database at Nepean hospital from 2006–2017. Participants were selected as depicted in Fig 1; following the prescribed criteria, the total study population consisted of 595 participants with LGA fetuses. The main analysis compared the subcutaneous fat of 353 obese, 145 overweight and 97 normal weight pregnant women; the second component of the main analysis reviewed 220 DM compared to 375 non-DM pregnant women. The sub-analysis assessed the effects of different DM types further dividing the sample groups (P-DM = 59, GDM-I = 132, GDM-D = 29 and non-DM/control = 375).

Maternal characteristics and fetal parameters were summarised in Table 1. Mean maternal age and median BMI were higher among DM mothers than controls. Neonatal birthweights were lower in the DM group however this was most likely due to the difference in average gestational age at time of delivery; on average neonates were delivered 7 days earlier compared to the control group. Birthweights taken at 38 weeks gestational age for standardisation demonstrated no significant difference between the means of the control and DM groups (4193.52 g (± 277.26) and 4143.82 g (± 384.75); p = 0.74). Groups were well matched for the remaining parameters.

**Table 1:** Baseline characteristics. Summary of maternal and fetal characteristics between DM and control participants.

		Total population	DM	Control	*p*-value
Participants (%)		595	220 (37%)	375 (63%)	
Maternal Characteristics	Age (mean [yrs] ± SD)	29.84 (± 5.74)	30.51 (± 6.02)	29.45 (± 5.54)	0.028*
	Height (mean [cm] ± SD)	167.15 (± 6.98)	166.6 (± 7.31)	167.48 (± 6.76)	0.138
	Pre-pregnancy BMI (median [kg/m^2^], IQ)	32.25 (26.83–38.01)	33.1 (27.91–38.51)	32.22 (26.08–37.72)	0.032*
	Parity (median, IQ)	1 (0–2)	1 (0–2)	1 (0–2)	0.071
	Smoking (%)	47 (8%)	22 (10%)	25 (7%)	0.194
BMI Group	Normal weight (%)	97 (16%)	28 (13%)	69 (18%)	0.72
	Overweight (%)	145 (24%)	49 (22%)	96 (26%)	
	Obese (%)	353 (59%)	143 (65%)	210 (56%)	
Diabetes Type	P-DM (%)	59 (10%)	59 (27%)		
	I-GDM (%)	132 (22%)	132 (60%)		
	D-GDM (%)	29 (5%)	29 (13%)		
Fetal Parameters	Delivery Age (mean [wks] ± SD)	38.49 (± 1.27)	37.85 (± 1.11)	38.86 (± 1.21)	<0.0001*
	Birth weight (mean [g] ± SD)	4259.93 (± 389.65)	4181.01 (± 414.23)	4306.23 (± 367.21)	<0.0001*
	>95th percentile (%)	421 (71%)	156 (71%)	265 (71%)	1
	Female (%)	195 (33%)	83 (38%)	112 (30%)	0.06

*Statistically significant (p < 0.05); DM, diabetes mellitus; P-DM, pre-gestational DM; I-GDM, GDM insulin managed and D-GDM, GDM diet managed

### Main analysis–

Table 2 shows the mean values of fetal abdominal and mid-thigh subcutaneous fat thickness at three time points representing early, middle and late stages of fetal fat development; the mean values were based on average weeks of gestation. The mean gestational age was 19.23 ± 0.68 weeks in the early time point, 28.98 ± 1.62 weeks in the middle time point and 36.20 ± 1.59 weeks in the late time point. Late time point measurements for AFT and TFT demonstrated a positive but weak correlation to birthweight. As expected, fetal fat measurements increased at each progressive time point. All fetal fat measurements in Table 2 were adjusted for gestational age. A comparison between BMI categories demonstrated no difference for measurements of AFT and TFT (p = 0.15 and p = 0.4, respectively). However, in women with DM, fetal AFT and TFT were significantly higher than controls; the largest difference was noted during the late time point (AFT– 0.55 mm and TFT– 0.53 mm; p < 0.0001).

**Table 2:** Main analysis BMI vs DM. Comparison between the effects of BMI and DM on fetal abdominal and mid-thigh fat. The baseline groups used for comparison were normal weight and the control group. Mean values were adjusted for gestational weeks.

Fat location	Category	GA adjusted mean in mm (95% CI)			Interaction *p*-value
		Early	Mid	Late	
Abdominal	Normal	1.45 (1.30–1.61)	3.20 (3.05–3.36)	5.67 (5.51–5.82)	0.15
	Overweight	1.39 (1.26–1.52)	3.23 (3.10–3.35)	5.39 (5.26–5.52)	
	Obese	1.39 (1.31–1.48)	3.19 (3.11–3.27)	5.60 (5.52–5.68)	
	Control	1.38 (1.30–1.46)	3.18 (3.10–3.25)	5.36 (5.28–5.43)	<0.0001*
	DM	1.44 (1.34–1.54)	3.24 (3.14–3.34)	5.91 (5.81–6.01)	
Thigh	Normal	1.26 (1.09–1.43)	3.31 (3.14–3.49)	5.51 (5.34–5.68)	0.4
	Overweight	1.30 (1.16–1.45)	3.38 (3.24–3.53)	5.54 (5.39–5.68)	
	Obese	1.35 (1.26–1.44)	3.41 (3.32–3.50)	5.76 (5.67–5.85)	
	Control	1.35 (1.26–1.44)	3.37 (3.28–3.45)	5.47 (5.38–5.56)	<0.0001*
	DM	1.28 (1.16–1.39)	3.43 (3.31–3.54)	6.00 (5.89–6.12)	

*Statistically significant (p < 0.05); GA, gestational age and DM, diabetes mellitus

The rate of fetal fat accretion was also assessed between BMI and DM categories. As seen in Table 3., fetal abdominal fat accrued faster in DM participants over time (p < 0.0001) with no difference noted between overweight and obese mothers (p = 0.34 and p = 0.39 respectively). Accelerated fetal mid-thigh fat accretion was also observed in DM participants (p < 0.0001) with no difference again seen between overweight and obese mothers (p = 0.81 and p = 0.06 respectively). Fetal fat in the DM mother was greatest during the mid and late stages of pregnancy for both the fetal abdomen (p < 0.0001) and mid-thigh (p < 0.0001). No difference for AFT was noted at the early stage between DM mothers and the control group (p = 0.09).

**Table 3:** Main analysis. Rates of fat accretion mm/week (95% CI) between maternal BMI categories and diabetes.

Fat location	Category	Early-Mid	p-value	Mid-Late	p-value	Early-Mid-Late	p-value
Abdomen	Normal	0.18 (0.16–0.19)		0.37 (0.34–0.40)		0.24 (0.23–0.25)	
	Overweight	0.19 (0.17–0.20)	0.47	0.32 (0.29–0.35)	0.019*	0.23 (0.22–0.24)	0.34
	Obese	0.19 (0.18–0.19)	0.39	0.34 (0.32–0.35)	0.08	0.25 (0.24–0.25)	0.39
	Control	0.18 (0.17–0.19)		0.32 (0.30–0.33)		0.23 (0.22–0.23)	
	DM	0.19 (0.18–0.20)	0.086	0.38 (0.36–0.40)	<0.0001*	0.26 (0.26–0.27)	<0.0001*
Thigh	Normal	0.20 (0.19–0.22)		0.32 (0.28–0.36)		0.24 (0.23–0.26)	
	Overweight	0.21 (0.20–0.22)	0.59	0.32 (0.29–0.35)	0.91	0.25 (0.23–0.26)	0.81
	Obese	0.21 (0.21–0.22)	0.29	0.33 (0.31–0.35)	0.75	0.26 (0.25–0.27)	0.06
	Control	0.20 (0.19–0.21)		0.30 (0.28–0.32)		0.24 (0.23–0.24)	
	DM	0.23 (0.22–0.24)	<0.0001*	0.36 (0.34–0.39)	0.0001*	0.28 (0.27–0.29)	<0.0001*

*Statistically significant (p < 0.05) and DM, diabetes mellitus

### Sub-analysis–

**Fig 2:**
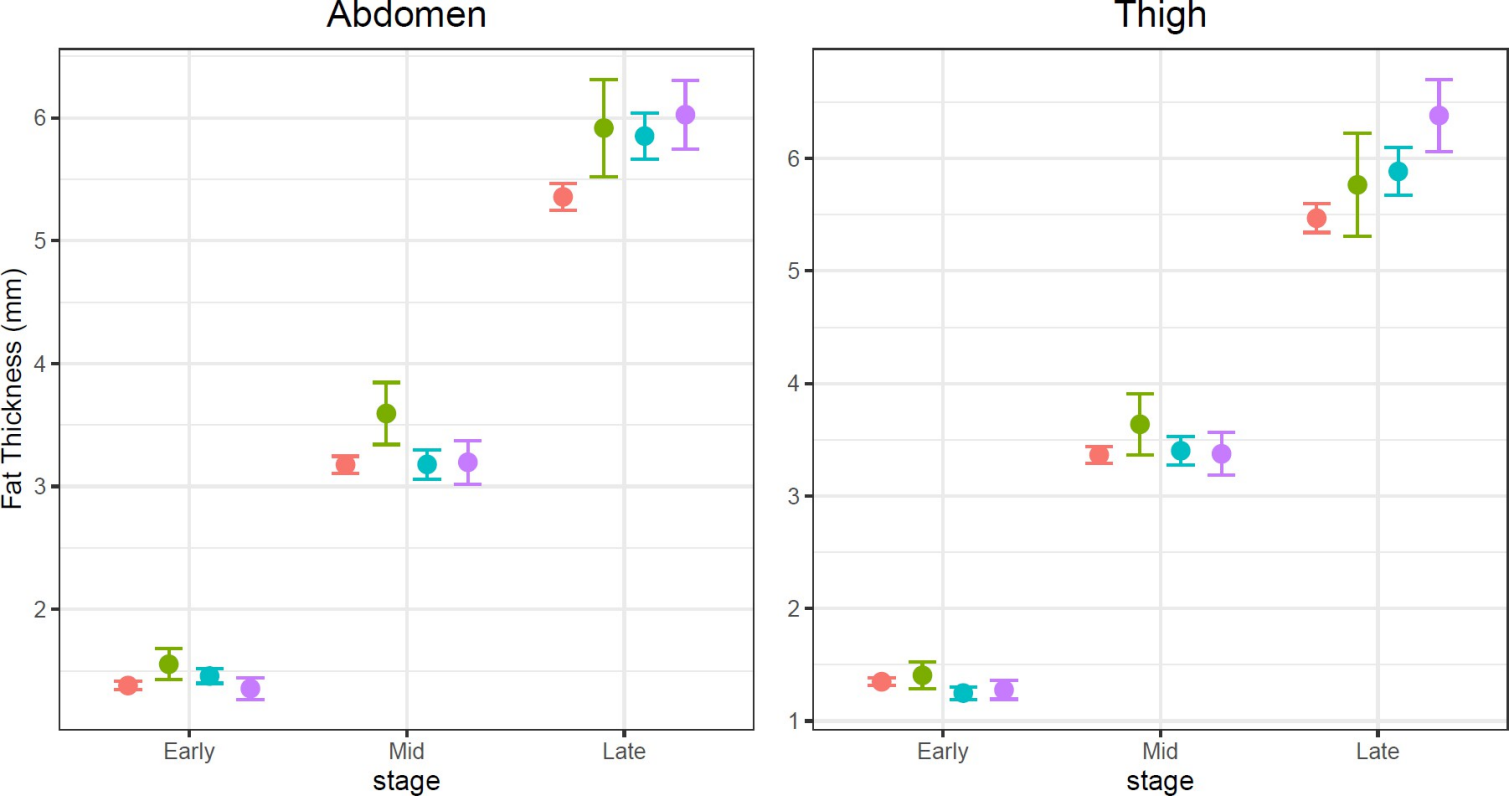
Subanalysis of different types of diabetes in pregnancy for the abdomen and mid-thigh. Mean fat thickness measurements are represented along the y-axis with early, middle and late stages of fetal fat accretion on the x-axis. Differences were noted for abdominal fat measurements at late stages of development in P-DM, I-GDM and D-GDM groups compared with the control (6.03 mm (5.75–6.30), p < 0.0001; 5.85 mm (5.67–6.04), p < 0.0001 and (5.92 mm (5.52–6.31), p = 0.0076, respectively). D-GDM also demonstrated early stage (1.55 mm (1.43–1.68), p = 0.0096) and mid stage (3.59 mm (3.34–3.85); p = 0.0018) differences. Mid-thigh fat differences were only seen in P-DM and I-GDM groups during the late 3^rd^ trimester scans (6.03 mm (5.75–6.30); p < 0.0001 and 5.85 mm (5.67–6.04); p = 0.001, respectively). Pink = Control; green = diet managed GDM; blue = insulin managed GDM and lilac = pre-gestational DM.

Different types of DM in pregnancy were analysed to determine the category with the greatest effect on abdominal and mid-thigh measurements. The time of subcutaneous fat accumulation, rate of growth and distribution was assessed for each DM subtype. As illustrated in Fig 2, AFT was markedly greater in all DM subtypes at the later stages of pregnancy (p < 0.001 across all types) than controls. The D-GDM group alone demonstrated an increase in AFT compared to the control group at each of the reported stages. TFT was notably thicker only in the P-DM and I-GDM groups (p < 0.001 and p = 0.001 respectively) compared with controls. No difference in TFT was seen in the D-GDM group compared with controls (p = 0.34) across all stages.

The rate of subcutaneous fat accretion for AFT was faster in all subtypes of the DM groups compared with controls when stages were pooled (p < 0.03; Table 4). Thigh fat accretion was faster in the P-DM (p < 0.0001) and I-GDM (p < 0.0001) participants but not in the D-GDM cohort (p = 0.18) across all stages. Overall, there was little effect from all DM subtypes on the AFT and TFT accretion rate during the early to middle stage of fetal fat development.

**Table 4:** Sub-analysis. Rates of accretion between different types of diabetes in pregnancy.

Fat location	Category	Early-Mid	p-value	Mid-Late	p-value	Early-Mid-Late	p-value
Abdomen	Control	0.18 (0.17–0.19)		0.32 (0.30–0.33)		0.23 (0.22–0.23)	
	P-DM	0.20 (0.18–0.21)	0.097	0.41 (0.37–0.45)	<0.0001*	0.28 (0.27–0.30)	<0.0001*
	I-GDM	0.19 (0.17–0.20)	0.48	0.37 (0.34–0.40)	0.00059*	0.26 (0.25–0.27)	<0.0001*
	D-GDM	0.20 (0.18–0.23)	0.11	0.34 (0.28–0.40)	0.39	0.26 (0.23–0.28)	0.025*
Thigh	Control	0.20 (0.19–0.21)		0.30 (0.28–0.32)		0.24 (0.23–0.24)	
	P-DM	0.22 (0.20–0.24)	0.036*	0.44 (0.39–0.49)	<0.0001*	0.31 (0.29–0.32)	<0.0001*
	I-GDM	0.23 (0.22–0.24)	0.00031*	0.34 (0.31–0.37)	0.038*	0.27 (0.26–0.29)	<0.0001*
	D-GDM	0.23 (0.20–0.25)	0.1	0.31 (0.24–0.38)	0.81	0.25 (0.23–0.28)	0.18

*Statistically significant (p < 0.05); P-DM, pre-gestational DM; I-GDM, GDM insulin managed and D-GDM, GDM diet managed

Overall, the diabetes group demonstrated increased AFT to TFT ratio compared to the control group, indicating raised abdominal fat deposition; this difference was observed across all stages of fat development (p < 0.001; Table 5). Further analyses of the DM subtypes did not detect differences between the control, P-DM and D-GDM groups, except for I-GDM. In relation to time points, the I-GDM group showed an increase in AFT to TFT ratio compared with controls at the early stages of fat development (p < 0.001) but not in the middle or late stages (p = 0.75 and p = 0.42 respectively).

**Table 5:** Fat distribution. Ratio of abdominal to mid-thigh for different types of diabetes in pregnancy.

Category	Early	p-value	Mid	p-value	Late	p-value	Pooled p-value
Control	1.07 (1.03–1.11)		0.97 (0.95–0.99)		1.00 (0.98–1.02)		
DM	1.21 (1.16–1.26)	<0.0001*	0.97 (0.94–1.00)	0.93	1.01 (0.98–1.04)	0.62	0.00049*
P-DM	1.16 (1.06–1.25)	0.11	0.97 (0.92–1.03)	0.82	0.97 (0.92–1.02)	0.31	0.36
I-GDM	1.25 (1.18–1.31)	<0.0001*	0.96 (0.92–1.00)	0.75	1.02 (0.98–1.05)	0.42	<0.0001*
D-GDM	1.13 (0.99–1.27)	0.42	0.99 (0.92–1.07)	0.5	1.05 (0.98–1.12)	0.21	0.16

*Statistically significant (p < 0.05); DM, diabetes mellitus; P-DM, pre-gestational DM; I-GDM, GDM insulin managed and D-GDM, GDM diet managed

The inter- and intra- variability between and within operators was calculated to determine the reproducibility of the measurements. A correlation coefficient based on pooled average AFT measurements showed excellent inter-operator and intra-operator agreement between BM (0.89) and PL (0.96) respectively. A strong correlation was also noted for thigh measurements (BM– 0.96 and PL– 0.98). The best reproducibility for both operators was seen at the mid and late stages of fetal fat development for AFT and TFT measurements.

## Discussion

The focus of this study was to explore the impact of maternal DM and obesity on subcutaneous fat in LGA fetuses. In our LGA cohort, fetal subcutaneous fat accretion increased with maternal DM but not with maternal BMI. During the 2^nd^ trimester, an increased AFT:TFT ratio was noted in fetuses of I-GDM women compared with controls but were similar by late 3^rd^ trimester.

This is the largest cohort of women with LGA babies to be studied for fetal subcutaneous fat thickness. In addition, this study measured AFT as a marker of central adiposity and TFT as a marker of peripheral fat to determine the ratio between these 2 measurements in the fetus.

Fetal subcutaneous fat has previously been used to estimate newborn body fat [28] and found to be associated with maternal diabetes [13, 20, 29]. Ratios of abdominal and leg fat in adolescents have been used to determine the risk for cardiometabolic disease (CMD) later in life [21]. Thus, it is reasonable to hypothesize that fetal subcutaneous fat may be valuable in predicting immediate and long-term metabolic complications.

Fetal subcutaneous fat thickness measurements directly correspond to neonatal fat thickness. Good correlation was noted between fetal and neonatal measurements from 172 participants (abdominal fat–r^2^ = 0.34 (p < 0.01) and femoral fat–r^2^ = 0.41 (p < 0.01)) [28]. In contrast to our study, Buhling et al. found no difference between GDM and control groups; however, it was noted that all patients had well managed GDM and the sample size was smaller. In the same study, maternal BMI was found to have no significant influence on sonographic abdominal measurements (p = 0.62), which is consistent with our findings (p = 0.15).

Maternal GDM, independent of fetal biometry, increases fetal anterior abdominal wall thickness (AAWT), as demonstrated by Aksoy et al. [20] and Venkataraman et al. [29]. AAWT was greater in 26 week fetuses from mothers with GDM (4.07 ± 0.46 mm) compared to the control group (3.28 ± 0.37mm; p < 0.01) in 176 women [20]. Venkataraman et al. assessed 331 participants at 20 weeks of gestation and reported AAWT measurements of 2.63 ± 0.51 mm for GDM mothers and 2.39 ± 0.41 mm for the control group (p < 0.01); at 32 weeks, AAWT measurements were 4.65 ± 0.81 mm for GDM mothers and 4.37 ± 0.66 mm for the control group (p < 0.01) [29]. The gestational ages used from both studies best represent the 3 time points from our study and our results demonstrated similar findings.

Tantanasis et al. demonstrated amongst 35 participants that fetal subcutaneous fat increased with abnormal OGTT results compared to controls (6.575 ± 0.993mm and 3.387 ± 0.613mm; p < 0.0005; respectively) [13]. A cut-off value of above 4.55 mm at 24–26 weeks gestation had 100% specificity and sensitivity for detecting abnormal GTT. It is important to note that their participants had a BMI of no greater than 30 kg/m^2^, thus showing maternal DM alone increased fetal subcutaneous fat accretion.

Cioffi et al. studied 3810 adolescents between the ages of 12–19 years to demonstrate that higher truncal to leg fat mass ratio (TLR), assessed by dual energy x-ray absorptiometry (DEXA), was positively associated with cardiometabolic risk factors in white and Mexican Americans [21]. Of note, DEXA cannot differentiate between abdominal visceral and subcutaneous fat mass components; however, studies have demonstrated that both are positively associated with cardiometabolic risk factors [14, 15]. Our assessment of fetal fat ratios between mothers with and without DM found early stage differences in fat distribution (p < 0.0001). Increased AFT:TFT ratio during the 2^nd^ trimester may be in response to early changes in the intrauterine environment associated with maternal DM. Although further investigation is required, intrauterine programming of fat distribution may increase the risk of CMD later in life.

In our study, maternal DM was associated with increased fetal subcutaneous fat accretion; however this was not seen amongst different BMI categories. A possible mechanism responsible for the underlying association would be that an excess of energy fuelled by glucose in DM mothers would result in the overdevelopment of fetal fat tissue. As supported by this study, mothers without excessive blood glucose demonstrated a significantly lower amount of fetal fat tissue compared to DM mothers. These changes in fetal subcutaneous fat measurements indicate that AFT and TFT are good screening tools to identify abnormal fetal fat development. These findings were also well supported by previously discussed studies. Subcutaneous fat measurements are relatively simple to obtain by appropriately trained healthcare professionals. The reported methods could be used in places where formal ultrasound scans are difficult to obtain, such as rural clinical practice, to screen for abnormal fetal growth.

Furthermore, the distribution of fetal fat was greater in the abdomen compared to the mid-thigh as seen in I-GDM mothers; this was noted prior to the detection of diabetes in the 2^nd^ trimester and before the commencement of treatment. It is important to monitor abdominal fat accretion as excessive centrally distributed fat is associated with CMD later in life [30]; whereas, peripheral subcutaneous fat provides a protective mechanism against metabolic dysfunction [16]. The increased fat ratio, caused by increase abdominal fat, noted in the 2^nd^ trimester may be a marker for early impaired maternal glucose levels indicating more severe GDM later in pregnancy whereby diet treatment may not be sufficient without insulin. Normalised fetal fat distribution in the third trimester time points, may be a response to stringent treatment plans provided after detection of diabetes. An extension to this study in comparing fetal fat to metabolic outcomes during adolescence may assist in identifying long term implications of maternal DM during pregnancy on offspring.

It is important to note that although statistically significant differences in fetal fat thickness were noted this may not be clinically significant if differences were small; significant increases in fat thickness ranged from 0.08 mm—0.67 mm. During the early stages of pregnancy, smaller differences between measurements may be difficult to reproduce. In clinical practice, late 3^rd^ trimester may be the only period where differences between groups are distinguishable.

Strengths of our study are the large number of participants with no missing data for the analysed groups and the fact that subcutaneous fetal fat measurements were highly reproducible between ultrasound operators.

Due to the retrospective nature of the study, limitations include lack of data regarding adequacy of glycaemic control (denoted by OGTT results), dietary intake and gestational maternal weight gain. A small group of participants from the last year of data collection (69 participants in 2017) were included using a newly adopted GDM management pathway, whereby OGTT threshold values were lowered to include mothers previously considered to be in the normal range; lowered differences in values for were 0.4 mmol/L at fasting and 0.5 mmol/L at 2 hours. A lack of glucose data entry made elimination of the glucose threshold variation for standardisation not feasible. Data on other possible confounders such as ethnic background, time of insulin introduction and insulin dosage were not obtained. Measurements of fetal fat were also limited by the availability of the archived images. It is also important to note that due to the time span of the study (11 years), the use of different operators in generating the images and changes to the machine models overtime, variability between studied images was expected. In order to overcome this issue, a single expert sonographer reviewed all cases to ensure the prerequisite anatomical landmarks were achieved before accepting the image for analysis. Cases were omitted whereby images did not satisfy the criteria for study measurements or images could not be measured with post processing software. The accuracy of the gestational age calculation and self-reported BMI was reviewed and confirmed by an appointed healthcare professional at the first antenatal clinic appointment to reduce the margin of error.

Maternal DM, independent of BMI, is associated with accelerated fetal subcutaneous fat accretion of the abdomen and mid-thigh in LGA fetuses. Increased fetal fat accretion occurs amongst different types of DM and following insulin treatment for GDM, the central to peripheral fat distribution ratio returns to normal proportions. This demonstrates that further study in the use of ultrasound to measure fetal fat distribution in predicting abnormal fetal growth is merited.

## Supporting information

S1 File (DOCX)

S2 File (XLSX)
