# Supplementary material for: Maternal diabetes independent of BMI is associated with altered accretion of adipose tissue in large for gestational age fetuses
Source: PLoS One. 2022 May 31;17(5):e0268972. doi: 10.1371/journal.pone.0268972 (PMC9154097; doi:10.1371/journal.pone.0268972)
Supplement: S1 File — (DOCX) [file pone.0268972.s001.docx]

Diabetes subtypes (mean - mm)

| **Fat location** | **Diabetic type** | **Early** | **E p-value** | **Mid** | **M p-value** | **Late** | **L p-value** | **EML p-value** |
| --- | --- | --- | --- | --- | --- | --- | --- | --- |
| Abdomen | Control | 1.38 (1.34-1.41) |  | 3.18 (3.11-3.25) |  | 5.36 (5.25-5.47) |  |  |
|  | P-DM | 1.36 (1.27-1.44) | 0.62 | 3.2 (3.02-3.37) | 0.83 | 6.03 (5.75-6.3) | <0.0001* | 0.14 |
|  | I-GDM | 1.46 (1.4-1.52) | 0.028* | 3.18 (3.06-3.3) | 0.96 | 5.85 (5.67-6.04) | <0.0001* | 0.08 |
|  | D-GDM | 1.55 (1.43-1.68) | 0.0096* | 3.59 (3.34-3.85) | 0.0018* | 5.92 (5.52-6.31) | 0.0076* | 0.066 |
| Thigh | Control | 1.38 (1.34-1.41) |  | 3.18 (3.11-3.25) |  | 5.36 (5.25-5.47) |  |  |
|  | P-DM | 1.36 (1.27-1.44) | 0.12 | 3.2 (3.02-3.37) | 0.93 | 6.03 (5.75-6.3) | <0.0001* | 0.077 |
|  | I-GDM | 1.46 (1.4-1.52) | 0.0024* | 3.18 (3.06-3.3) | 0.63 | 5.85 (5.67-6.04) | 0.0011* | 0.31 |
|  | D-GDM | 1.55 (1.43-1.68) | 0.37 | 3.59 (3.34-3.85) | 0.059 | 5.92 (5.52-6.31) | 0.22 | 0.34 |

*Statistically significant (p < 0.05), P-DM, pre-gestational DM; I-GDM, GDM insulin managed and D-GDM, GDM diet managed
